# Supplementary material for: Iridophores as a source of robustness in zebrafish stripes and variability in Danio patterns
Source: Nat Commun. 2018 Aug 13;9:3231. doi: 10.1038/s41467-018-05629-z (PMC6089994; doi:10.1038/s41467-018-05629-z)
Supplement: Supplementary file 1 — Supplementary Information [file 41467_2018_5629_MOESM1_ESM.pdf]

Supplementary Material for "Iridophores as a source of robustness in zebrafish stripes and variability in *Danio* patterns" by Volkening and Sandstede

Supplementary Methods: Detailed Simulation Conditions

The parameters in Supplementary Tables 1-4 and Supplementary Fig. 18 apply to all our simulations except for the mutant- or experiment-specific adjustments specified below. All simulations begin with the same initial condition at 21 dpf on a domain 2000 μm long $\times$ 1000 μm high (unless noted below): a uniform random distribution of $N_{\text{init}}^{\text{M}}$ melanophores and $N_{\text{init}}^{\text{X}}$ loose xanthophores, and a single strip of dense iridophores spaced 50 μm apart at the center of the domain. Domain growth rates are 38 μm per day lengthwise and 27 μm per day in height with the single exception of Supplementary Fig. 13c. To make reproducing our results easier, we gather all adjustments to these simulation conditions below:

- *Pfeffer*: Turn off all X^{d} an X^{l} birth by setting $N_{\text{rand}} = N_{\text{init}}^{\text{X}} = 0$ (see Supplementary Fig. 5).
- *Nacre*: Turn off all M birth by setting $N_{\text{init}}^{\text{M}} = N_{\text{diff}} = 0$ (see Supplementary Fig. 6).
- *Shady*: Remove I^{d} cells from the initial condition. Frohnhöfer *et al.*[1] describe xanthophores appearing in X0 at a delayed time in *shady*, so we also introduce a strip of X^{d} (100 μm apart) at the center of the domain at 22 dpf (see Supplementary Fig. 7).
- *Pfeffer*;*nacre*: Implement both *pfeffer* and *nacre* changes (that is, set $N_{\text{init}}^{\text{M}} = N_{\text{diff}} = N_{\text{rand}} = N_{\text{init}}^{\text{X}} = 0$) (see Supplementary Fig. 9d).
- *Pfeffer*;*shady*: Turn off X^{d} and X^{l} birth and implement *shady* adjustments described above (see Supplementary Fig. 9b).
- *Nacre*;*shady*: Turn off M birth and implement *shady* changes described above (see Supplementary Fig. 9e).
- *Pfeffer* with earlier iridophore placement (Supplementary Fig. 5d): Informed by[1], we begin our simulations at an earlier time point in development with a smaller domain (811 μm high $\times$ 1734 μm long). The initial condition at this point is a single strip of I^{d} 50 μm at the center of the domain; xanthophore birth is off ($N_{\text{rand}} = N_{\text{init}}^{\text{X}} = 0$); M birth is off initially ($N_{\text{init}}^{\text{M}} = N_{\text{diff}} = 0$), but at 21 dpf we return $N_{\text{diff}} = 350$.
- *Choker*: Initial condition lacks strip of I^{d}. We add 15 I^{d} cells at randomly selected locations on the domain 10 days after the simulation begins at 31 dpf (see Fig. 6f and Supplementary Fig. 12).
- *Puma*: M birth is reduced by setting $N_{\text{diff}} = 50$. (See Fig. 6d and Supplementary Fig. 13b).
- *Puma* with reduced growth (Supplementary Fig. 13c): M birth is reduced by setting $N_{\text{diff}} = 50$ and domain growth rates are reduced by 67%.
- Ablation Experiment 1[2] (Fig. 6b): Simulation begins under normal wild-type conditions. Remove all M, X^{d}, and X^{l} cells, as well as 20% each (randomly selected) of the I^{d} and I^{l} cells, in a 1000 μm wide region in the center of the domain (spanning the full domain height) at 40 dpf.
- Ablation Experiment 2[2] (Supplementary Fig. 14b): Simulation begins under normal wild-type conditions. Starting at 41 dpf and continuing for 7 days, remove all M and X^{l} cells, as well as 20% (randomly selected) of I^{l} cells, in a 1000 μm wide region in the center of the domain in the regions of stripes 1V and 1D (the vertical positions and spans of these stripes are approximated based on our wild-type simulations).
- Ablation Experiment 2[2] (Supplementary Fig. 14c): Simulation begins under normal wild-type conditions. Starting at 41 dpf and continuing for 7 days, remove all M, X^{l}, and I^{l} cells in a 1000 μm wide region in the center of the domain in the regions of stripes 1V and 1D (the vertical positions and spans of these stripes are approximated based on our wild-type simulations).
- Iridophore Ablation Experiments (Supplementary Figures15b & 15c): Initial condition is altered so there is a break in the central strip of I^{d} at 21 dpf.
- Temperature-sensitive *pfeffer* (Supplementary Fig. 16b): Simulation begins under normal wild-type conditions. At 65 dpf, remove all X^{d} and X^{l} from the domain and halt their further birth by setting $N_{\text{rand}} = 0$.

- Reduced iridophore dynamics (Figures 7-8): Note that, because of the form of the inequalities (Equations 3-6) governing iridophore dense-loose transitions, changing the parameters c, d, e, f, g, h (which all represent numbers of cells in a given neighborhood) to unrealistically large or negative numbers effectively turns off different components of our dual rules (see Fig. 5), producing the reduced conditions in Figures 7 & 8. The parameters changed for each figure are as follows: $g = 1000$ X^{d} cells (and/or $h = -1$ X^{d} cells) in Fig. 7a; $f = 1000$ M cells in Figures 7b & 7h; $g = -1$ X^{d} cells in Fig. 7c; $c = 1000$ M cells in Fig. 7d; $e = 1000$ X^{d} cells in Fig. 7e; $d = 1000$ X^{d} cells (and/or $e = -1$ X^{d} cells) in Figures 7f & 7g; $f = 1000$ M cells and $g = -1$ X^{d} cells in Fig. 7i; $d = -1$ X^{d} cells in Fig. 8b; $c = 1000$ M cells and $d = -1$ X^{d} cells in Fig. 8c; $h = 1000$ X^{d} cells in Fig. 8e; $f = 1000$ M cells and $h = 1000$ X^{d} cells in Fig. 8f.

- Alternative xanthophore dynamics (Supplementary Fig. 17): Remove signal from X^{d} to X^{l} to become dense by setting $p = 0$.

Supplementary Note 1: Melanophore Survival Signal

Nakamasu *et al.*[3] showed that M depend on long-range signals from interstripe cells for survival, and we initially assumed this signal could be provided by X^{d} or I^{d}. This allowed us to explain *pfeffer* and *shady*: if the survival signal came only from X^{d} or only from I^{d}, respectively, we would have continual M death in both these phenotypes. The temperature-upshift experiment[4] (Supplementary Fig. 16) complicated the picture, however: removing xanthophores from the fish skin later in development after stripes form (using a temperature-sensitive *pfeffer* allele) clearly results in M death, and our model could not explain this behavior when we assumed I^{d} provide a survival signal to M. Instead, the long-range survival signals from iridophores maintained wild-type patterns without xanthophores in our simulations. We saw this effect in our temperature-sensitive *pfeffer* simulations because the density of M and I^{d} was normal when xanthophores were removed from the fish skin (in contrast, M density is reduced under normal *pfeffer* conditions because xanthophores promote M birth). This suggests that the hypothesized long-range promotion of M by I^{d} is too strong a signal for fully formed patterns, and, hence, that the long-range survival signals deduced in[3] are the result of xanthophore-melanophore interactions alone. Without long-range survival signals from I^{d}, it was unclear how to account for *pfeffer*, and we thus introduced an additional component to our M death rules. We specify that blue I^{l} atop M offer weak local support, partially maintaining M even when X^{d} are not present at long-range. Alternatively, this rule could be indirectly capturing signals from L-iridophores, which appear below M and I^{l} after patterning and are not included in our model; this supports suggestions by Frohnhöfer *et al.*[1].

Supplementary Note 2: Alternative Xanthophore Dynamics

During wild-type patterning, loose xanthophores across the skin seem to respond to local cues, becoming dense in response to the appearance of I^{d} and appearing loose in black regions[5,6]. Recall that, with the addition of a signal from X^{d} to X^{l}, our model rules for xanthophore form changes (given by Equations 1-2) support these findings. In particular, X^{l} become dense when the sum of interstripe cells nearby is greater than the corresponding sum of stripe cells (note that X^{d} are only factored into the interstripe sum $p = 50\%$ of the time). Furthermore, in contrast to our nonlinear iridophore dynamics, which seem to necessarily include long-range interactions to account for the presence of orange spots in *nacre*, here we predict xanthophore form is guided entirely by local communication. This supports experimental suggestions[6] that cell form involves gap junction-dependent interactions.

A recent study[6] (post initial model development) complicates the xanthophore picture and may be related to the signal we added from X^{d} to X^{l}: Mahalwar *et al.* found that xanthophores cannot be sharply categorized into loose and dense; instead, intermediate forms are found in the *shady* and *leopard* mutants[6]. During model development, the *shady* mutant was the phenotype we struggled most to account for while simultaneously producing wild-type and the other mutants lacking cell types. The work[5,7–9] had established that I^{d} reach prospective interstripe locations and then induce loose xanthophores, spread across the skin, to become dense. Without directions from iridophores, it is unclear how to account for *shady*, and we were unsure what our target phenotype should be: should *shady* have xanthophores in loose or dense form (or a combination of both)? This question is further complicated by the lack of experimental images of the

double mutant *nacre*;*shady* (only xanthophores). Without images of *nacre*;*shady*, it is unclear what form xanthophores self-select to appear in (changing the parameter p controls this in our simulations).

Based on the distinct black spots present in *shady*[1] (as well as a brief early 2013 statement, without data shown, that xanthophores appear in a dense sheet in *shady*;*nacre*[1]), we made the modeling choice to produce *shady* phenotypes with X^{d}. Without X^{d}, there is nothing present to gather melanophores into spots. The question then became how to signal X^{l}, present across the skin, to become dense without the deduced signals from iridophores[8,7,5]. We address this by introducing the added signal from X^{d} to X^{l} in Equation 2: each X^{l}, with p = 50% probability each day, responds to local signals from its dense neighbors to become dense. In effect, this means X^{d} are weakly preferred over the loose form, and will win out on *shady*;*nacre*. Without this addition, we obtain the alternative images in Supplementary Fig. 17, which feature a mix of xanthophores in both loose and dense forms in *shady* and *shady*;*nacre*. We consider this mixture of xanthophore forms to be one way our model, with two distinct classes of xanthophores, could account for the intermediate xanthophores now known to be present in *shady*[6]. Because removing our signal between xanthophores (setting $p = 0$) does not impact wild-type, *nacre*, or *pfeffer*, we thus present it as an alternative candidate model.

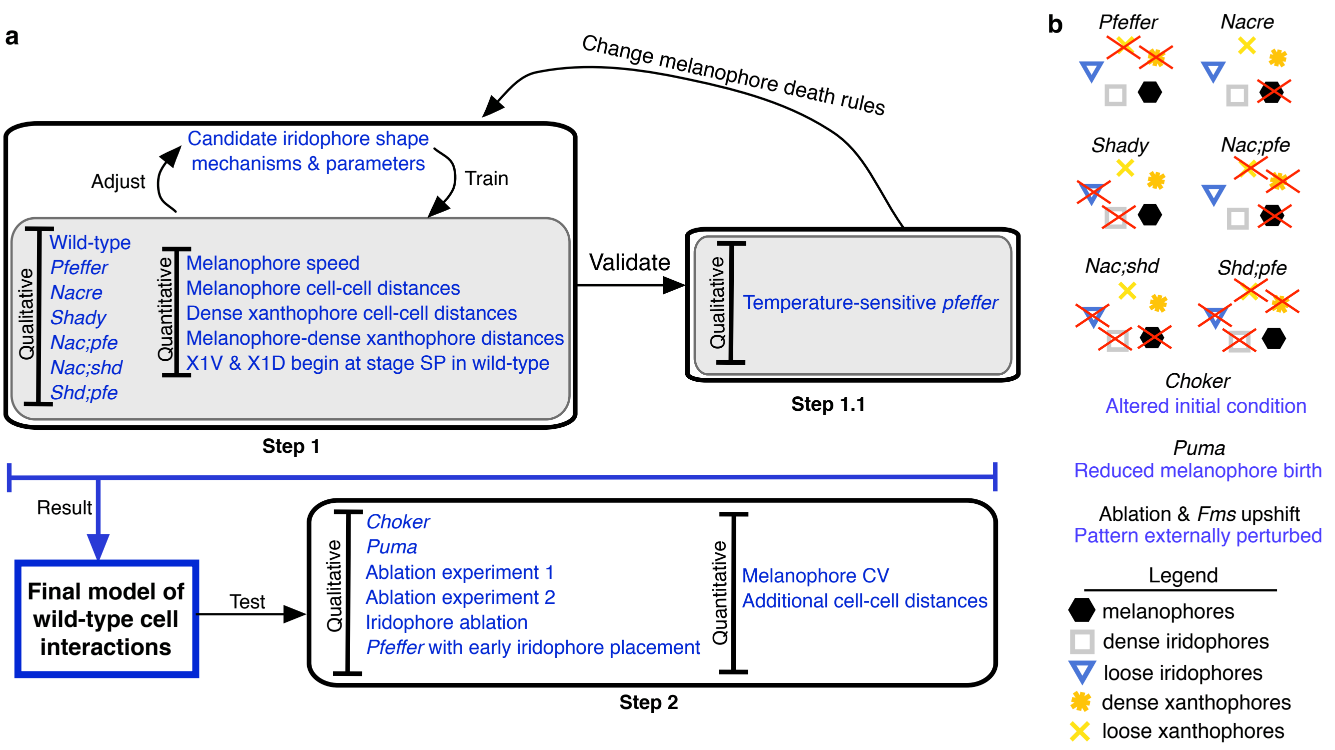

Supplementary Figure 1: Modeling approach and set-up. (a) Flowchart showing modeling approach and output. The majority of work went into Step 1, which was repeated many times to account for current unknowns about iridophores. Qualitative studies of *pfeffer* and *nacre* motivated candidate iridophore form-transition mechanisms, while quantitative measurements were used to help calibrate parameters and length-scales to the biology. We identified the proposed iridophore interaction network by requiring consistency across wild-type and mutants lacking cell types (and associated double mutants). At one point (Step 1.1) in Step 1, simulating a temperature-sensitive *pfeffer* allele[4] led us to reframe our rules for melanophore death (see Supplementary Note 1). After this side-step to Step 1.1, we continued to fine-tune in Step 1. The result is a model of wild-type cell interactions that is consistent with the experimental set-ups and quantitative measurements in the flowchart. (b) Summary of simulation conditions for mutants lacking cell types, *choker*, *puma*, and experiments. These dynamics are accounted for using the same set of cell interactions, and the only changes are described pictorially: mutants that lack pigment cells are simulated by turning off birth of the cells impacted (motivated by Frohnhöfer *et al.*[1], *shady* also involves the delayed addition of X^d at the horizontal myoseptum); the *choker* mutant phenotype arises due to an altered initial condition; the *puma* mutation reduces melanophore birth; and ablation and temperature experiments are simulated by externally perturbing the pattern or altering growth rates.

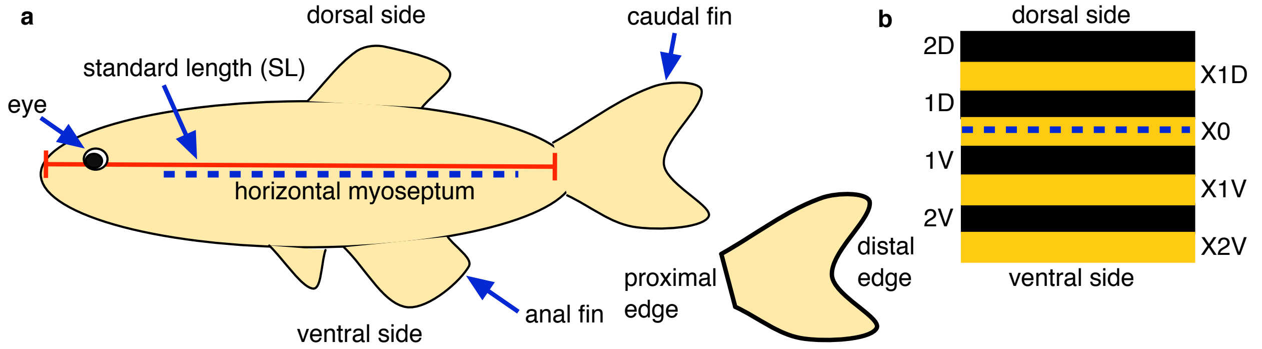

Supplementary Figure 2: Summary of zebrafish anatomy and measurements related to this work. (a) Zebrafish anatomy. The horizontal myoseptum at the center of the fish body helps align the pattern horizontally[1]. Standard length (SL) is a measurement of the body length, and standardized standard length (SSL) is a measure of average body length based on reference zebrafish[10]. (b) Black stripes and yellow interstripes are labelled outward from the horizontal myoseptum toward the dorsal and ventral edges of the fish, as X0, 1D and 1V, X1V and X1D, 2D and 2V, and so on[1].

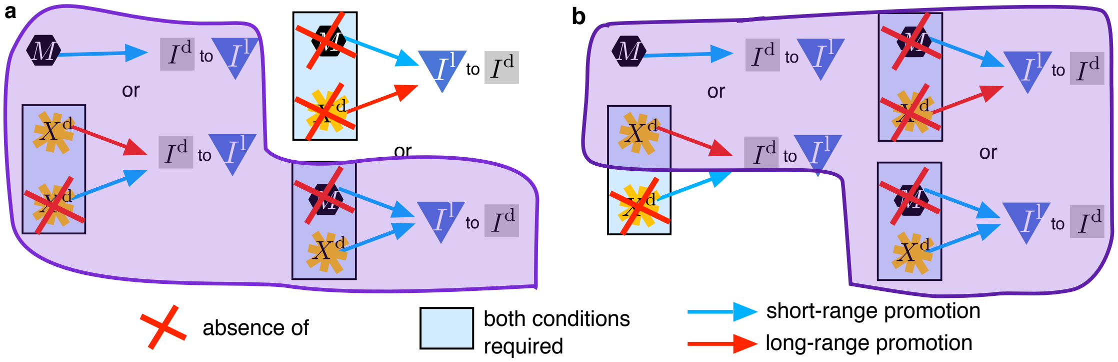

Supplementary Figure 3: Simplified iridophore form-change mechanisms consistent with other *Danio* fish. (a) To simulate the patterns observed on *D. albolineatus*[11] (Fig. 8), we simplify the proposed network of iridophore interactions on zebrafish: purple highlighted interactions show the portion of iridophore signals on zebrafish that our model suggests are active on *D. albolineatus*. (b) Similarly, purple highlighted region indicates the iridophore interaction pathways we suggest *D. margaritatus* has in common with zebrafish. These altered networks indicate where gain-of-function mutations may have altered cellular interactions to produce zebrafish patterns during *Danio* evolution.

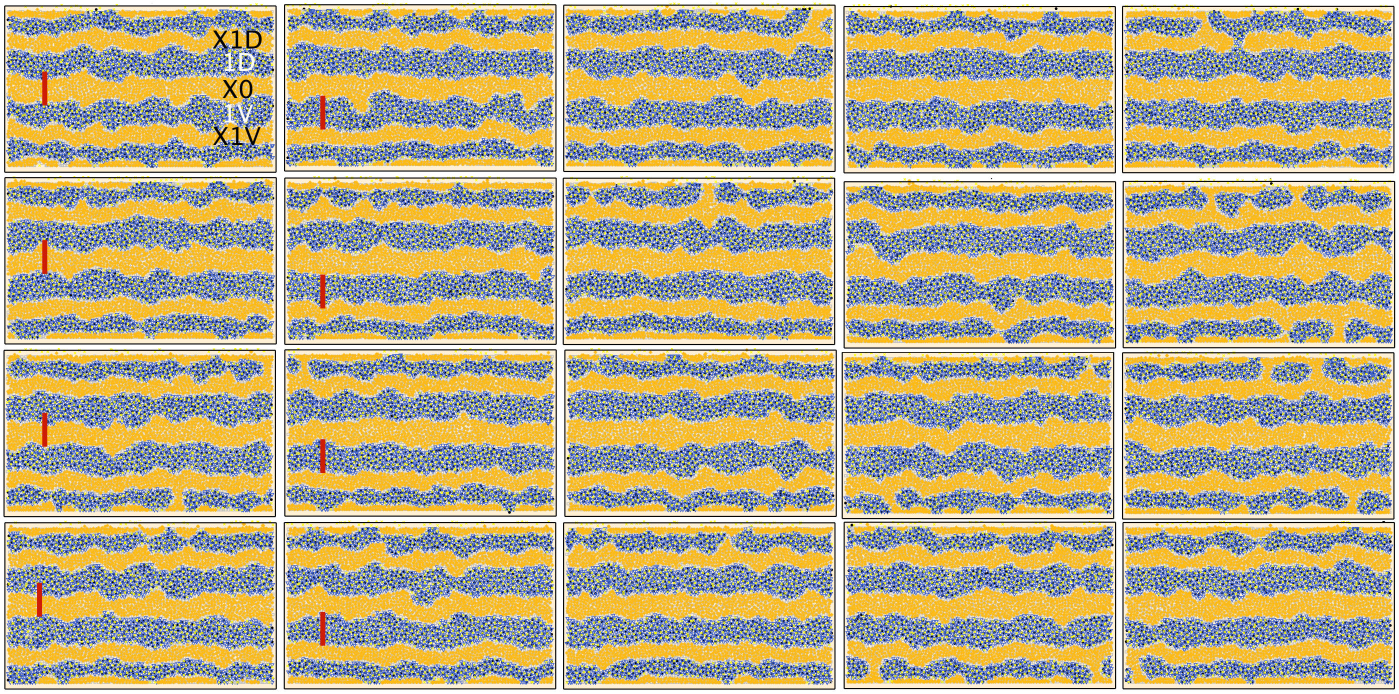

Supplementary Figure 4: Additional examples of wild-type results, shown at stage J+ (roughly 71 dpf). Vertical scale bars (500 μm) are provided to visualize pattern width. Based on 100 simulations, we find that 11% feature small breaks in interstripes (e.g. a bridge connecting to black stripes): in 4% this break occurs in X1V, in 6% it is present in X1D, and in one simulation we find a break in X0 (we do not count breaks in the interstripes at the upper and lower boundaries of the domain). For animations of wild-type development, see Supplementary Movies 1 & 2.

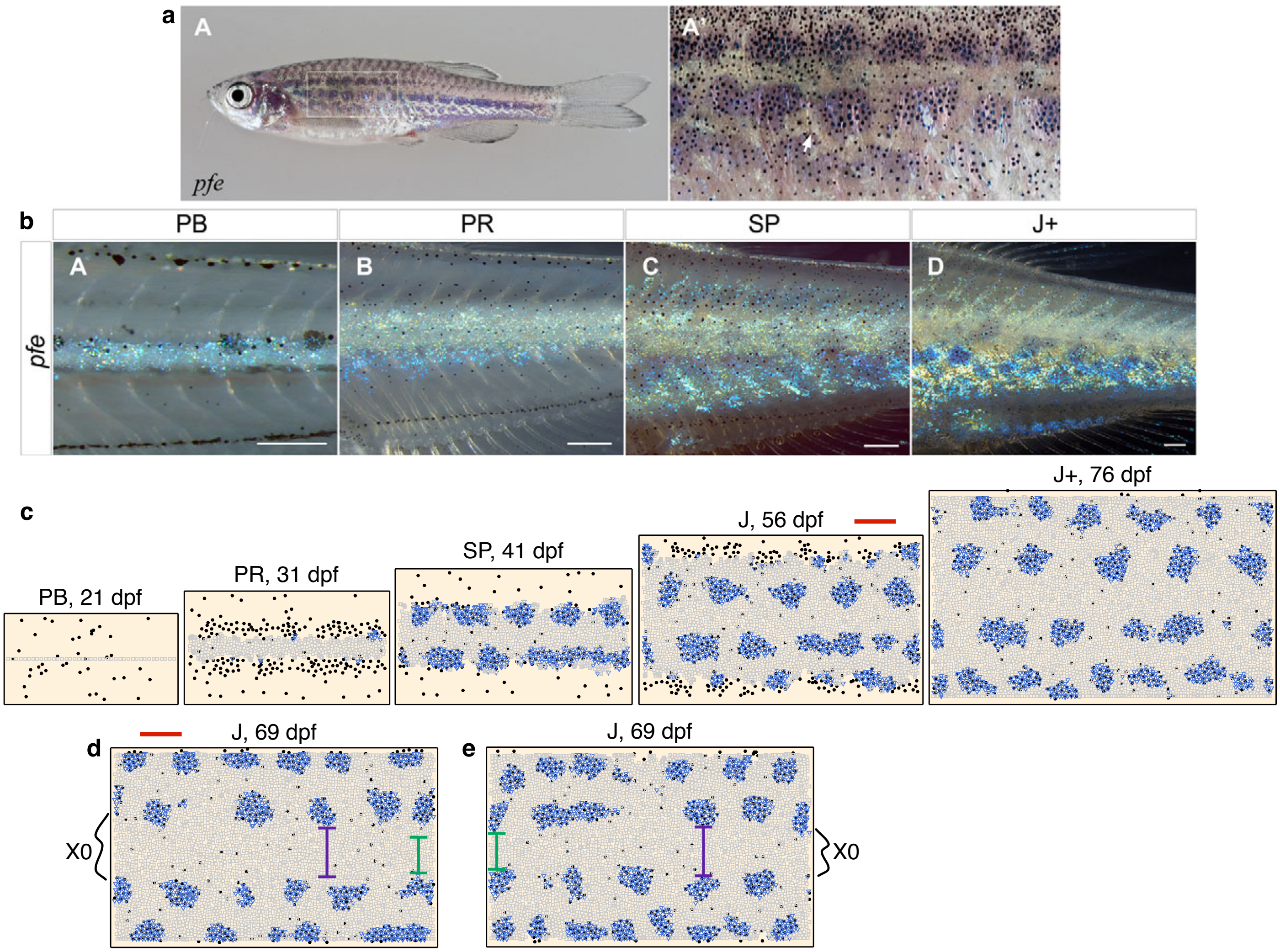

Supplementary Figure 5: *Pfeffer*. (a) *Pfeffer* zebrafish from[1]. (b) Timeline of *pfeffer* development (scale bar: 250 μm). (c) Simulated *pfeffer* development. Also see Supplementary Movie 3. (d-e) Occasionally $I^{\rm d}$ appear along the horizontal myoseptum early; when this occurs, the central X0 region occupied by silver $I^{\rm d}$ is enlarged[1]. (d) We test this by simulating pattern formation from stage CR (in this case, informed by Parichy *et al.*[10], our initial domain is 811 μm high and 1734 μm wide). Here we specify the appearance of $I^{\rm d}$ at the horizontal myoseptum as our initial condition and hold M differentiation off until 7 days later at 21 dpf, the normal starting point of our simulations. (e) A normal *pfeffer* simulation for easier comparison with *pfeffer* under earlier $I^{\rm d}$ appearance. To compare length scales, bars are provided: green bars are the same length in both figures, as are the longer purple bars. As shown, the central silver region appears slightly expanded under earlier iridophore placement, but additional statistics on our simulations, as well as empirical data quantifying the difference in X0 widths under these 2 settings, would need to be collected to test this conclusively. Scale bar is 500 μm in all simulated images. Empirical images (a-b) are reproduced from Frohnhöfer *et al.*[1] and licensed under CC-BY 3.0 (http://creativecommons.org/licenses/by/3.0); published by The Company of Biologists Ltd.

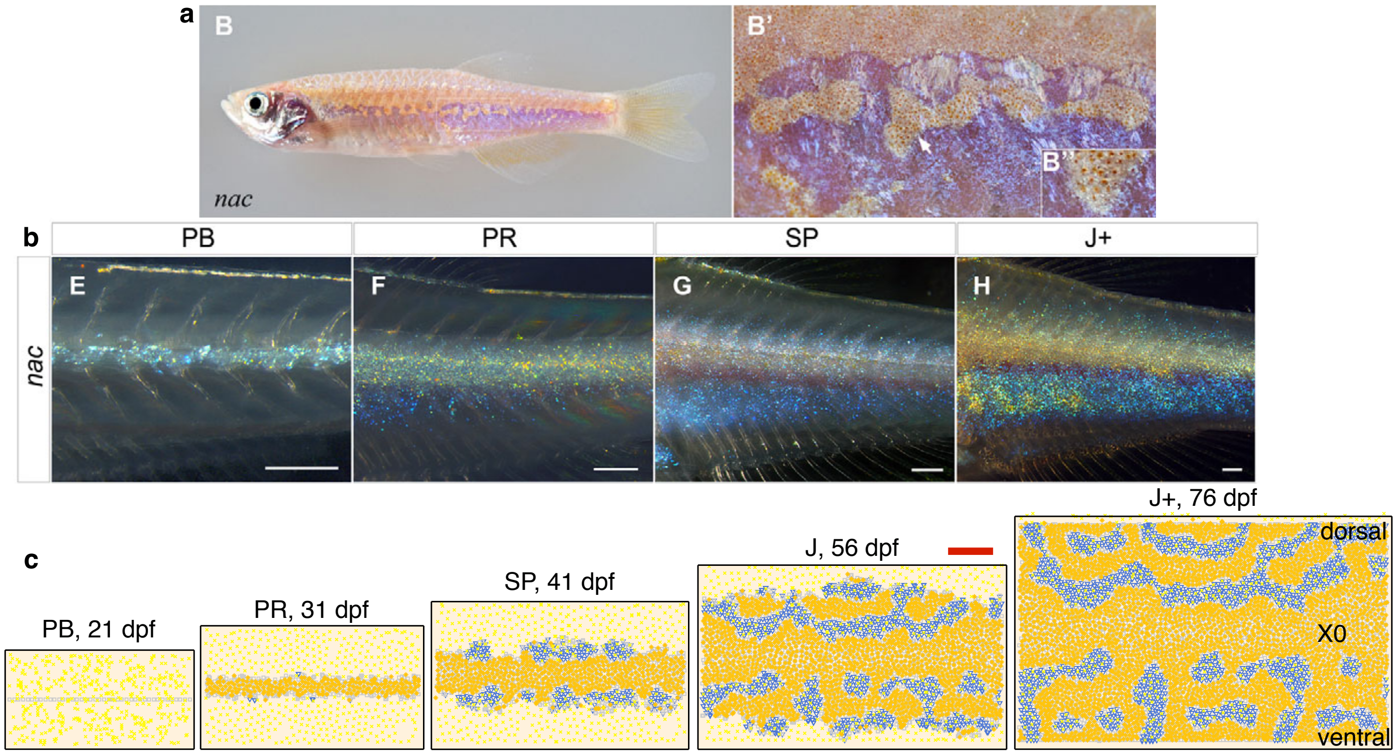

Supplementary Figure 6: *Nacre*. (a) *Nacre* fish from[1]. (b) Timeline of *nacre* development[1] (scale bar: 250 μm). (c) Simulated *nacre* development (scale bar: 500 μm). We see an expanded X0 region with messy borders, with a combination of orange and blue spots away from the horizontal myoseptum. Note that, because we do not make any distinction between ventral and dorsal dynamics in terms of domain growth or cell interactions, we cannot account for differences in these two regions. This is a simplifying assumption, and our simulations expectedly produce the same kind of patterns across the domain. Empirical images (a-b) are reproduced from Frohnhöfer *et al.*[1] and licensed under CC-BY 3.0 (http://creativecommons.org/licenses/by/3.0); published by The Company of Biologists Ltd.

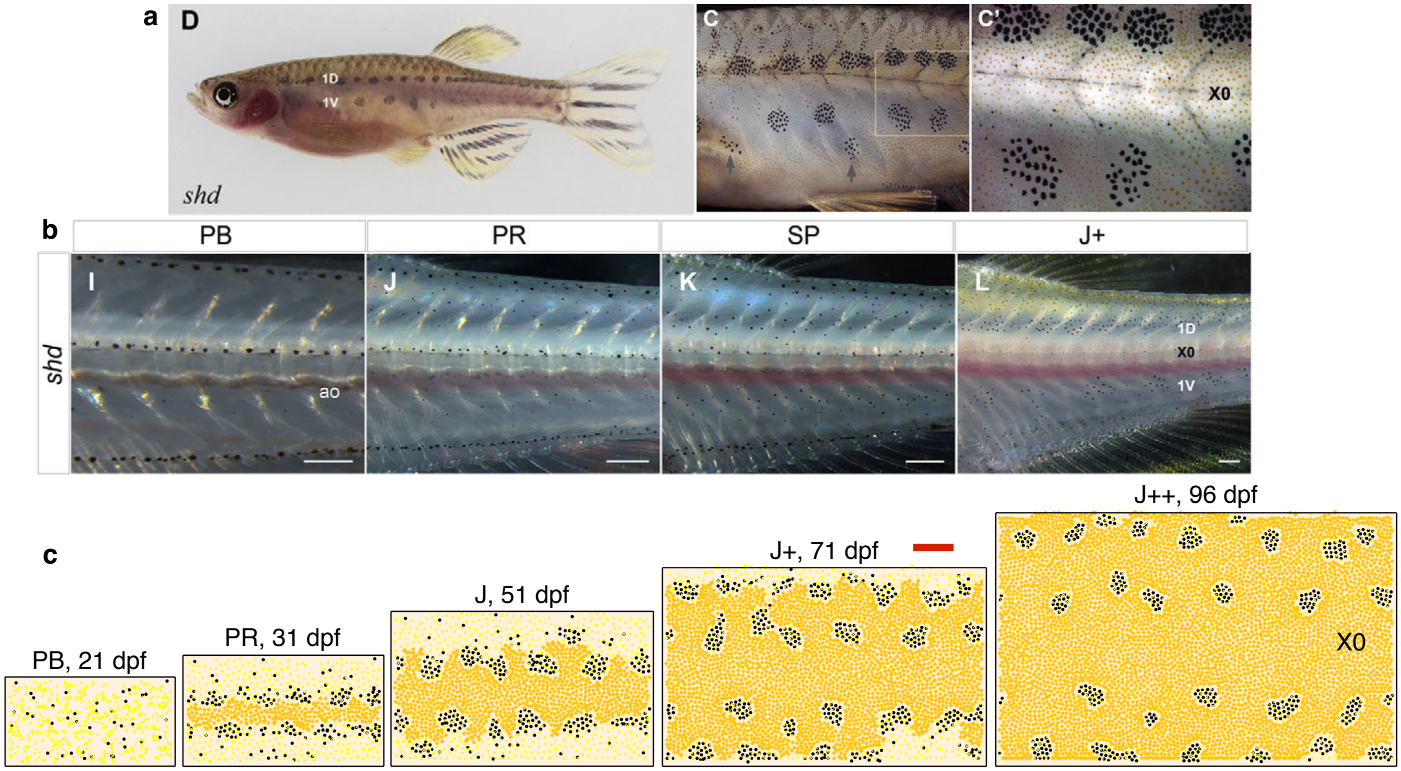

Supplementary Figure 7: *Shady*. (a) *Shady* fish[1]. (b) Timeline of *shady* development[1] (scale bar: 250 μm). (c) Simulated *shady* development (scale bar: 500 μm). *Shady* (*shd*) is variable and depends on the strength of the mutation. Notably, while *shady* is characterized by clean black spots of melanophores across an expanded xanthophore background, 20% of *shady* fish have a random distribution of melanophores instead of spots ventrally[1]. The black spots often seem to be arranged in strips dorsal and ventral to the X0 region. Because *shady* lacks iridophores, we simulate this mutant without the strip of dense iridophores in our initial condition. Frohnhöfer *et al*. describe xanthophores appearing in X0 at a delayed time. Thus, we place a strip of X^d in the center of the domain at 22 dpf. Empirical images (a-b) are reproduced from Frohnhöfer *et al.*[1] and licensed under CC-BY 3.0 (http://creativecommons.org/licenses/by/3.0); published by The Company of Biologists Ltd.

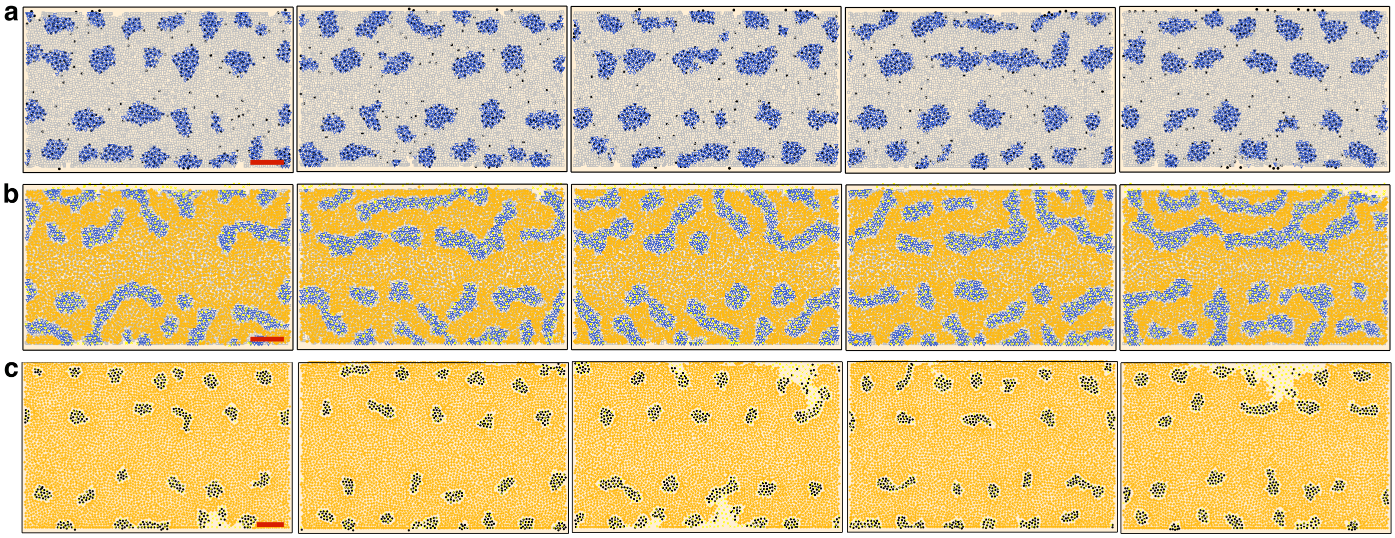

Supplementary Figure 8: Additional sample simulations of *pfeffer*, *nacre*, and *shady*. Images show the pattern variability produced by our model. *Pfeffer* and *nacre* are shown at stage J+ (roughly 71 dpf). *Shady* is shown at stage J++ (96 dpf). Scale bars are 500 μm.

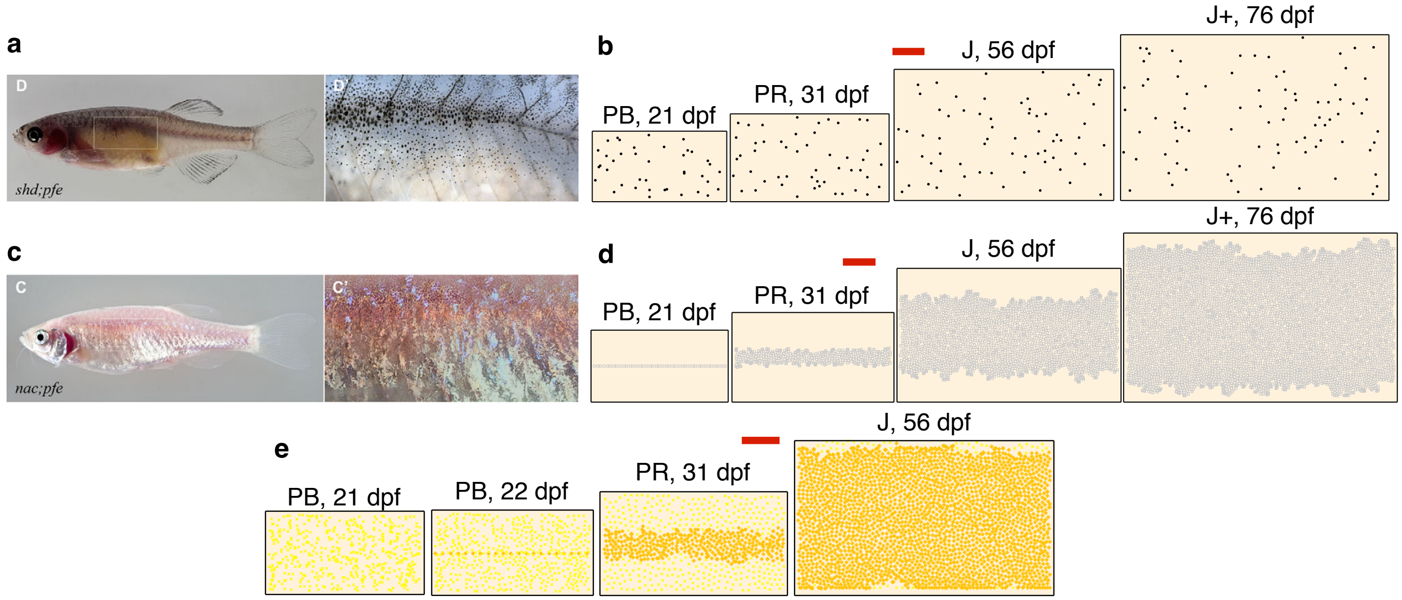

Supplementary Figure 9: Double mutations in *nacre*, *pfeffer*, or *shady*. These fish feature only one of the 3 main cell types: melanophores, xanthophores, and iridophores[1]. (a-b) Empirical image of *shady*;*pfeffer* (only melanophores) and simulated development. (c-d) Empirical image of *nacre*;*pfeffer* (only iridophores) and simulated development. (e) Simulated development of *nacre*;*shady* (only xanthophores; experimental image not available). Simulation scale bars are 500 μm. Note that our *shady*;*pfeffer* features uniform melanophore distributions, instead of heavier density near the horizontal myoseptum, because we introduced no preference for location of melanophore precursors into our model. One way to account for the appearance of more melanophores near the center of the fish in *shady*;*pfeffer* would be to use a different distribution for selecting random locations for black cell birth. Fish images (a) and (c) are reproduced from Frohnhöfer *et al.*[1] and licensed under CC-BY 3.0 (http://creativecommons.org/licenses/by/3.0); published by The Company of Biologists Ltd.

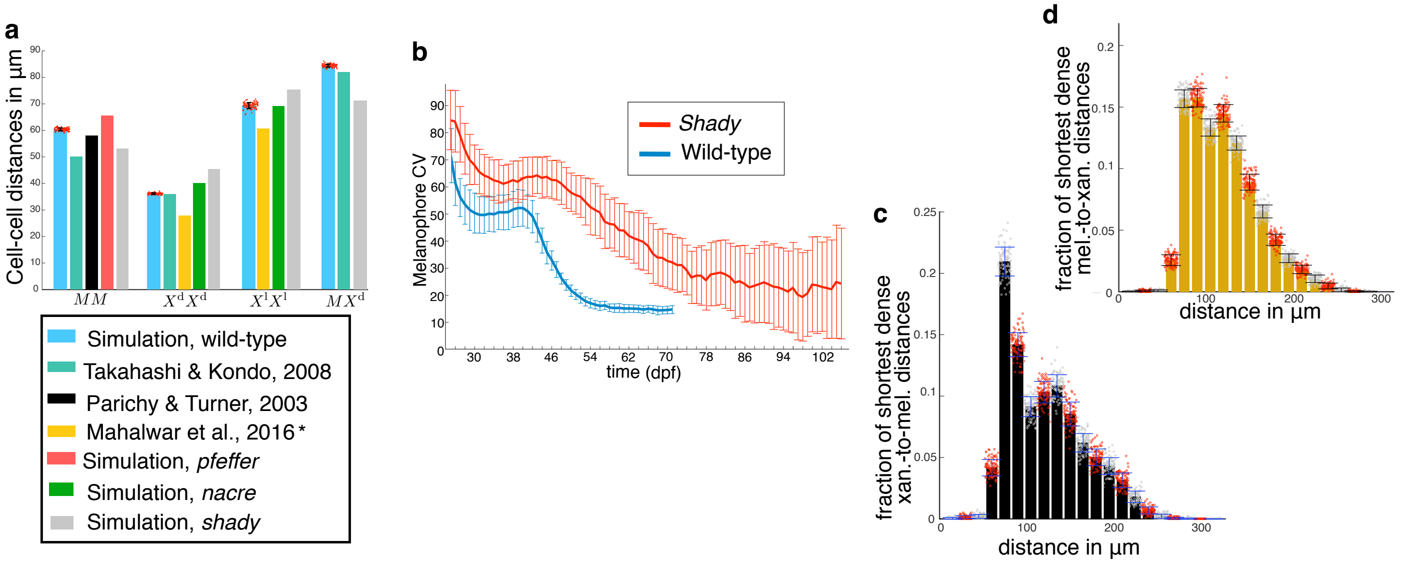

Supplementary Figure 10: Quantitative measurements on cell distances. (a) Nearest neighbor distances. Simulated distances are reported at J+ (71 dpf) in wild-type, *nacre*, and *pfeffer*, and at J++ (96 dpf) in *shady*. Empirical measurements are approximations of data from[12,13,6]. Note that the distances reproduced from Mahalwar *et al.*[6] are for xanthophores with and without iridophores (not strictly xanthophores in dense and loose form, as presented here). Mahalwar *et al.*[6] showed that the xanthophores in *shady* are in an intermediate form (in contrast, *nacre* features dense xanthophores atop dense iridophores). In particular, *shady* xanthophores are more spread out than dense xanthophores in wild-type. As shown, our model produces xanthophores that are slightly more spread out in *shady* than in wild-type, in agreement with[6]. (b) Coefficient of variation (CV = 100 $\times$ standard deviation/mean) for the distance between neighboring melanophores for wild-type and *shady* (see Fig. 6g in the main text for *pfeffer*). CV is a local measurement of pattern quality[4,13]. Low CV values are associated with better formed patterns and decay during wild-type patterning from roughly 100 to 30 (based on our approximation of data in[13]). Shown are average values across simulations with standard deviation. The high standard deviation in *shady* CV values may reflect the high variability of *shady* phenotypes[1]. (c) The average distribution of shortest distances to dense xanthophores for each melanophore provides information on stripe width (data shown at stage J+, 71 dpf)[14]. To calculate the distribution for each simulation, we find the closest xanthophore to each melanophore on the domain, bin these distances in 15 μm wide bins, and scale by the number of melanophores present to produce a distribution. We then take the average of these distributions across 100 wild-type simulations to obtain the average distribution. (d) Similarly, average distribution of shortest distances to melanophores for each dense xanthophore is related to interstripe width (data shown at stage J+, 71 dpf). We caution that these distributions provide a lower estimate on pattern width, as newer stripes and interstripes at the edges of our domain are much narrower than the older pattern at the center of the fish (vertical scale bars are provided in the wild-type simulations in Supplementary Fig. 4 as an alternative means of quickly gauging pattern width). Note that stripes and interstripes grow with age (an observation we made from[10]); stripes on adult fish are roughly 600 μm wide[15], while adult interstripes are on the order of 400 μm wide[16] (adult is the stage after J++[10]). Wild-type statistics are based on 100 simulations, *nacre* and *pfeffer* on 80 simulations, and *shady* on 50 simulations. Error bars denote standard deviation and overlaid scatter points represent all observations used to construct the bar charts.

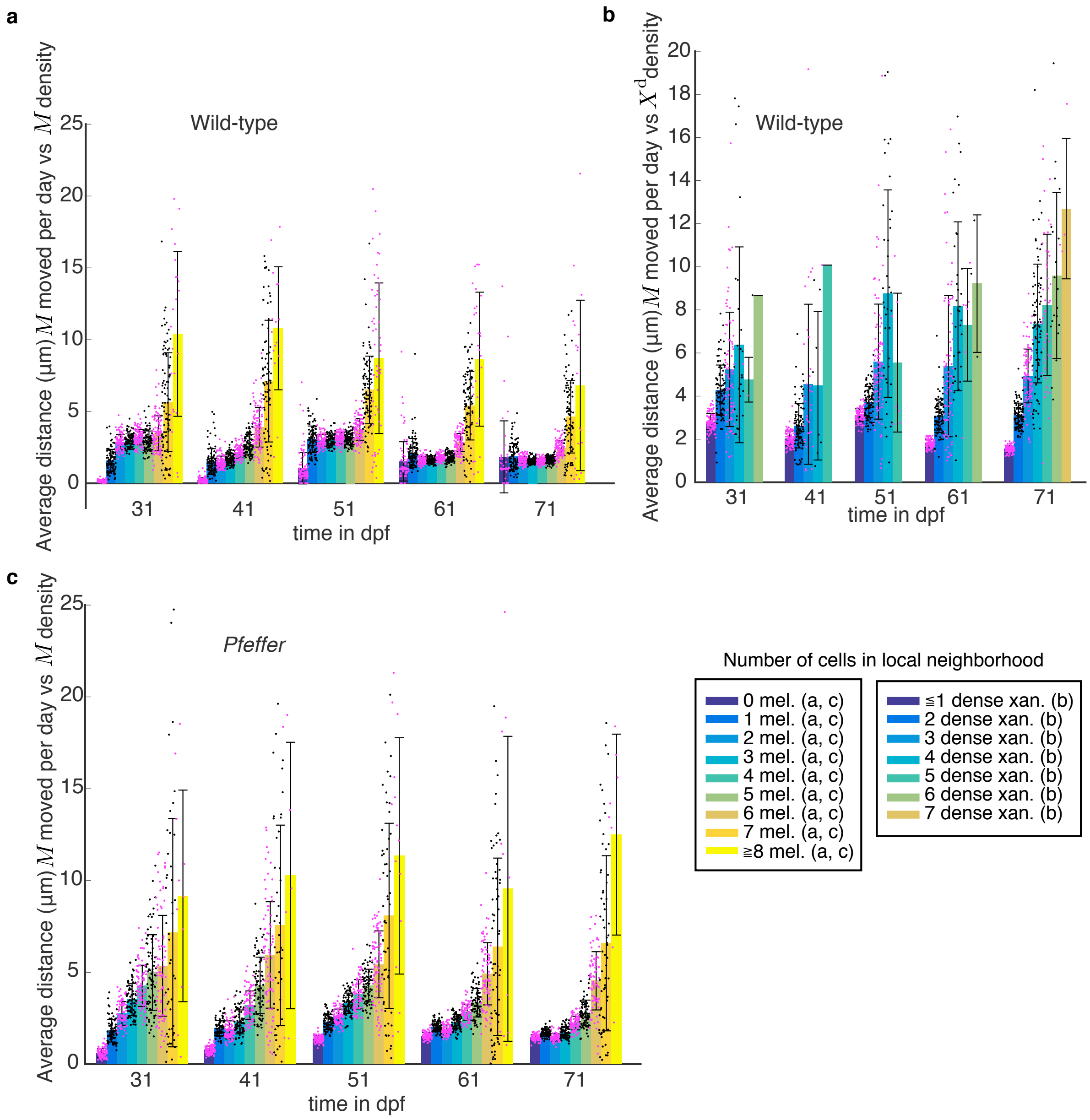

Supplementary Figure 11: Statistics on melanophore migration. We show average distance moved per day in μm at 5 different sample times in development (given in dpf, days post fertilization; note our simulations begin at 21 dpf). Different bars distinguish between different environments (the number of cells in a Δ_{xm} neighborhood surrounding the moving cell, see legend). Note that distance moved is due to migration alone (after the previous cell positions are stretched due to growth, we calculate the difference in position pre- and post- migration step). Data on melanophore speeds varies in the experimental literature. Takahashi *et al*. report melanophores moving 80-100 μm in one week during patterning *in vivo*[12]; Walderich *et al*. describe melanophores as typically differentiating from precursors near their final position and moving little[17]. Our melanophore speeds are based on[12], and, while individual black cells in our simulations do move on the order of 15 μm per day, migration is very sensitive to local cell neighborhood. Averages and standard deviation (denoted by bars) are computed using 100 simulations for wild-type and 80 simulations for *pfeffer*; overlaid scatter points represent all observations used to construct the bar charts.

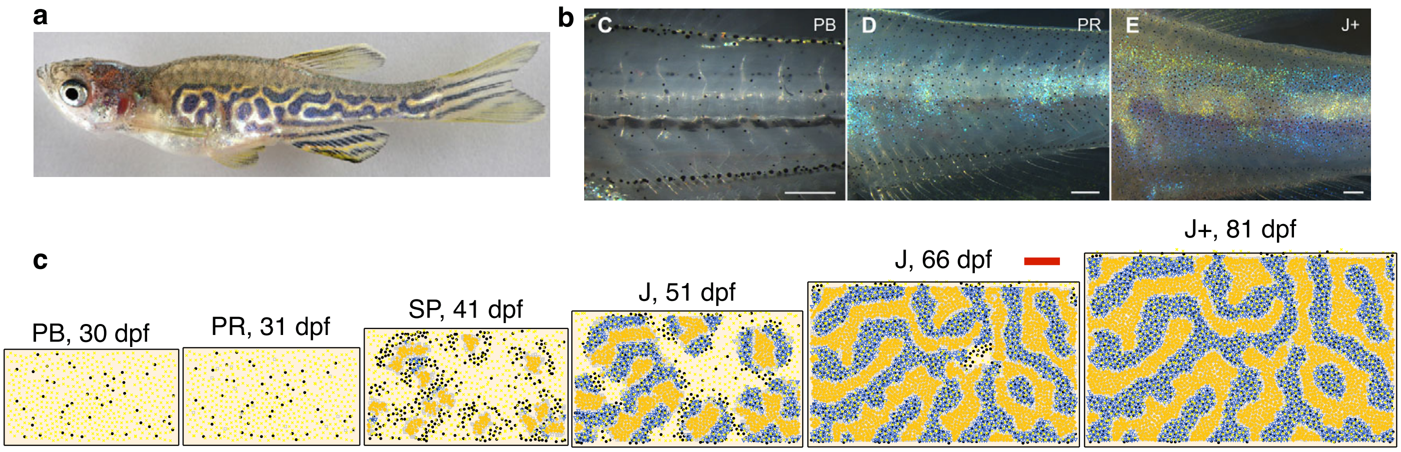

Supplementary Figure 12: *Choker* zebrafish. The *choker* zebrafish lacks a horizontal myoseptum (and the strip of dense iridophores associated with it), leading to labyrinth-style patterns[1]. (a) Example *choker* fish. (b) *Choker* development (scale bar: 250 μm); melanophores appear randomly across the body at stage PB, but iridophores do not emerge until stage PR, and, when they do so, they arise in patches with arbitrary locations[1]. Frohnhöfer *et al.* describe xanthophores appearing within the new iridophore patches at stage PR. (c) Simulated *choker* development (note: motivated by[1], I^{d} are not present in our initial condition at 21 dpf, but instead 15 I^{d} are introduced at random locations at stage PR). Scale bar is 500 μm. We stress that no changes are made to our wild-type parameters in any way: cell interaction rules, as well as the number of random locations evaluated for M birth per day, are kept constant. Also see Supplementary Movie 6. Images (a) and (b) reproduced from Frohnhöfer *et al.*[1] and licensed under CC-BY 3.0 (http://creativecommons.org/licenses/by/3.0); published by The Company of Biologists Ltd.

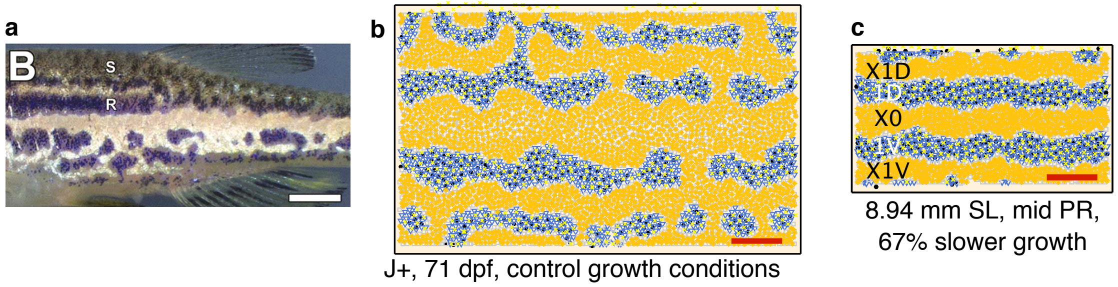

Supplementary Figure 13: *Puma* zebrafish. The *puma* mutant features heavily reduced melanophore birth[18,13]. (a) *Puma* mutant (scale bar: 2 mm); image reproduced from Parichy *et al.*[18] with permission from Elsevier; Copyright (2003) Elsevier Science, U.S.A. (b) *Puma* phenotype simulation (note this image is repeated from Fig. 6 in the main text for comparison). We simulate *puma* by simply reducing the number of random locations selected each day for possible melanophore birth (from 350, used throughout our other simulations, to 50). Biologically, this could correspond to reducing the number of melanophore precursors in the skin. (c) *Puma* under reduced domain growth. Parichy and Turner[13] showed that raising *puma* fish in conditions associated with reduced growth rates led to wild-type patterns. To simulate this condition, we reduce our growth rates by 67% (the developmental stages associated with the resulting domain sizes are then approximated using SSL from Parichy *et al.*[10]). In comparison to (b), slowing growth can reclaim wild-type stripes (though interstripes X1V and X1D form more quickly, relative to the domain size, in comparison to our wild-type simulations). Simulation scale bars are 500 μm.

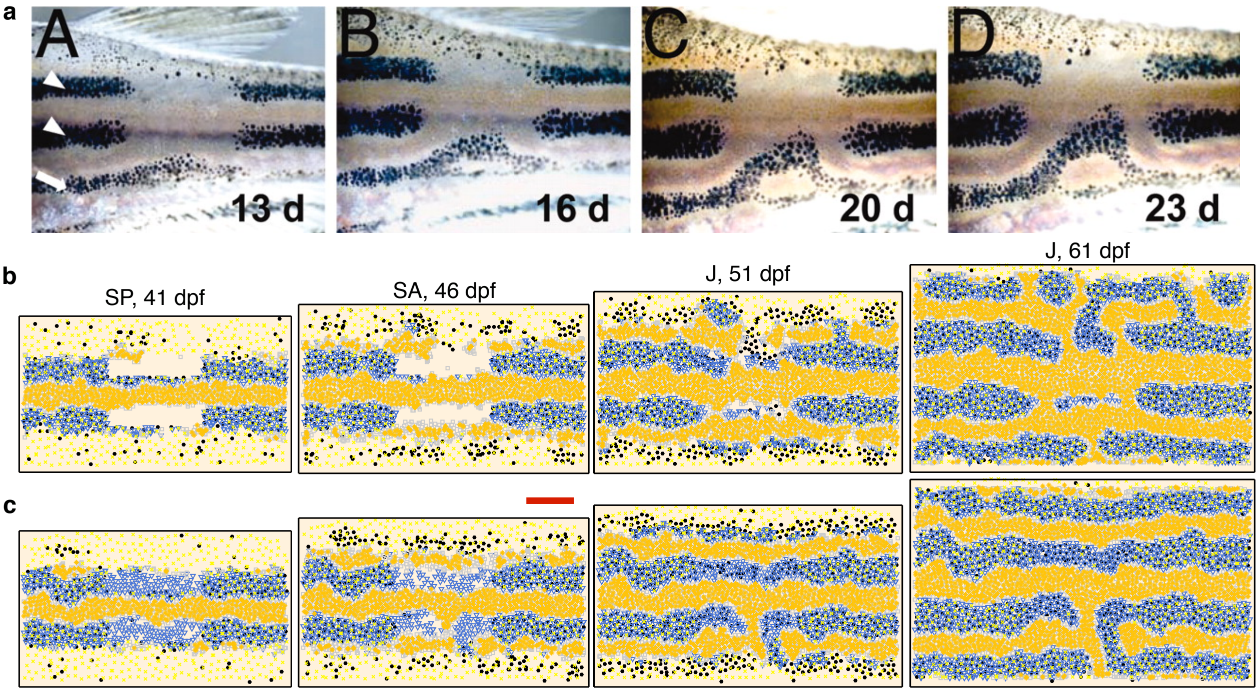

Supplementary Figure 14: Experiment 2[2]: continual ablation of melanophores in stripes 1V and 1D for one week. (For Experiment 1[2], see Fig. 6a in the main text). (a) Example timeline of ablation[2] (time is given in days (d) post ablation); in this set-up, Yamaguchi *et al*. continually ablated melanophores in stripes 1V and 1D for a week, while other pigment cells were not purposely harmed[2]. This experiment was done on 3 zebrafish, and the resulting dynamics led to stripe 2V (white arrow) moving upward to produce a downward parabola shape or connecting with 1V to form a bridge across the X1V interstripe (two fish, results presented as SI animations in[2]). We note that scale bars, stages, and zebrafish ages are not present or reported for this experiment[2], making it more challenging to determine the conditions. (b-c) Simulated Experiment 2 (scale bar is 500 μm): we begin removing melanophores in the area of stripes 1V and 1D (in a 1000 μm long region) at 41 dpf. (The vertical span of stripes 1V and 1D used for this ablation experiment is approximated based on our wild-type simulations). We continue removing black cells each day in these regions for 7 days; because laser ablation disrupts pigmented cells[19], we also remove all loose xanthophores in these regions each day. In a recent article[19], Watanabe and Kondo clarified that laser ablation also impacts iridophores, killing roughly 20% of these cells below xanthophores. We thus test two options for the impact of the laser on iridophores, either (b) removing all loose iridophores in the ablated region, or (c) killing off a randomly selected 20% each day. In these example simulations, we observe stripe 2V connecting to 1V or stripe 2D bridging across X1V to connect with 1D. Though qualitatively similar to the corresponding dynamics on the fish skin[2] (more so to the animations in the SI of[2] than the figure provided), we consider these results not as clean as our work with Experiment 1 (Fig. 6a-b). This could provide a place for model improvement or be a by-product of the complicated nature of this experiment; continued laser ablation of cells across 7 days may have other impacts on the skin not accounted for. Image (a) reproduced from Yamaguchi *et al.*[2] with permission; Copyright (2007) National Academy of Sciences, U.S.A.

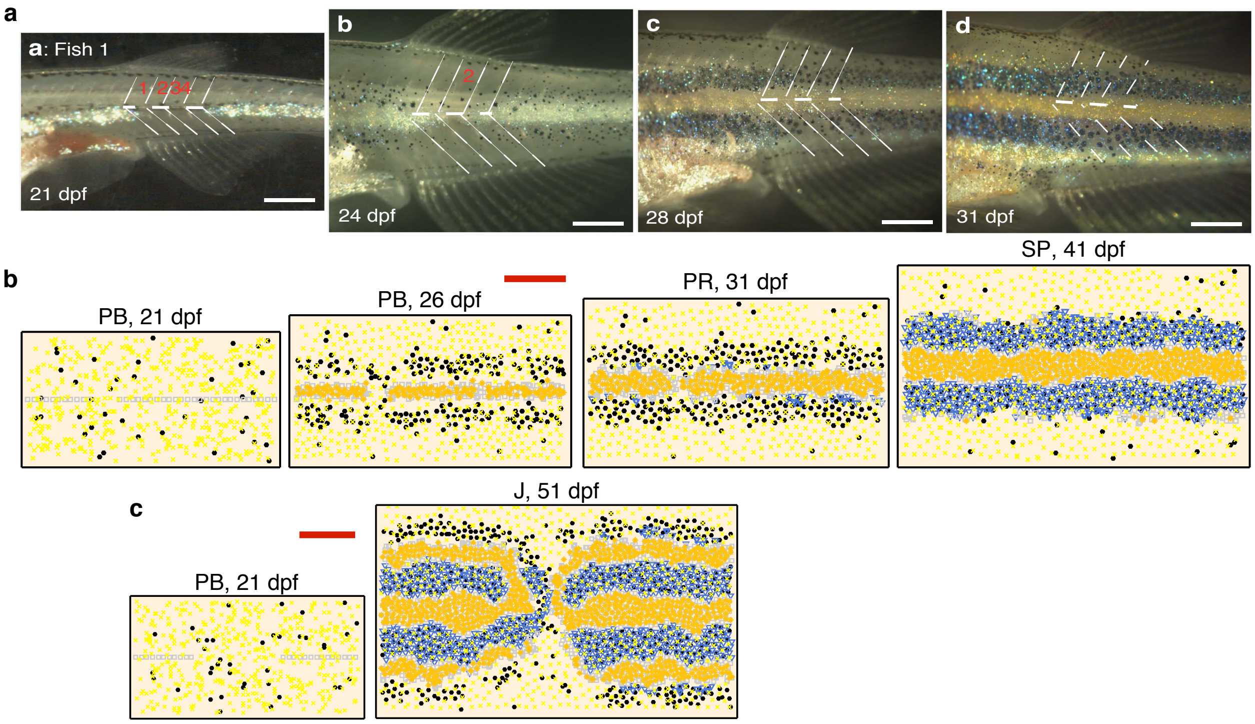

Supplementary Figure 15: Iridophore ablation[17,7]. (a) Ablation of I^{d} using an ErbB inhibitor in a small gap at the center of the fish by Walderich *et al.*[17] does not impact final patterning (note this type of ablation may also impact melanophores[20,21]). (b) Simulated ablation of iridophores in a small gap. (c) Simulated ablation of iridophores in a larger gap at 21 dpf disrupts stripe alignment (compare to images of ablation of iridophores by Patterson and Parichy[7]; this ablation[7], using nitroreductase, more strongly impacts final patterning and causes melanophores to wrap around remaining interstripes). Note that our time points (in dpf) do not match completely with those in the empirical timelines, and we expect this is because our growth rates are slower. Growth rates differ significantly depending on lab conditions, as well as on stochastic and genetic effects[10,22]. Empirical scale bars are 250 μm in (a); simulation scale bars are 500 μm. Image (a) reproduced from Walderich *et al.*[17] and licensed under CC-BY 4.0 (http://creativecommons.org/licenses/by/4.0).

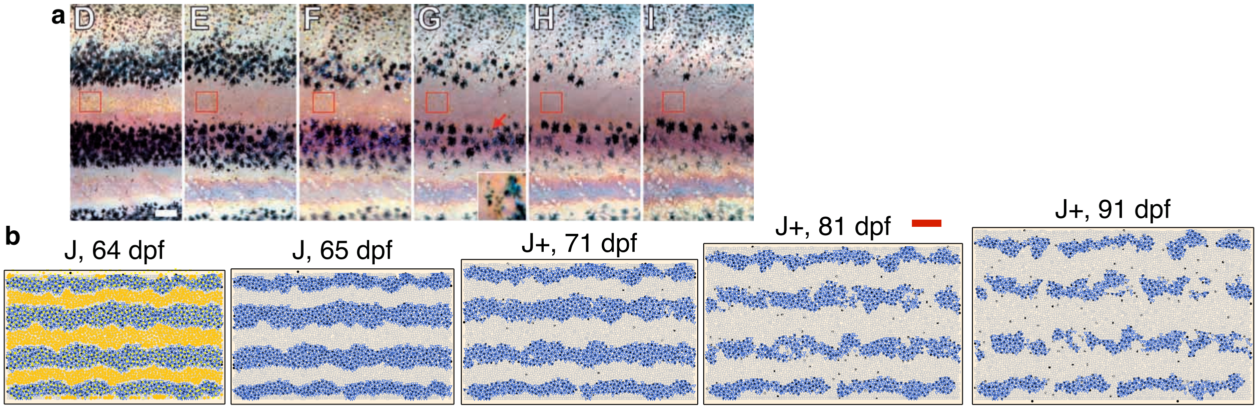

Supplementary Figure 16: Temperature-sensitive fms. Increasing the temperature of the environment for the temperature-sensitive fms mutant essentially transforms the fish from wild-type to *pfeffer* (no xanthophore) conditions and allows experimentalists to tune when xanthophores disappear[4]. (a) Temperature upshift experiment on the fish skin, leading to gradual stripe degeneration[4]; scale bar is 250 μm. (b) Simulated upshift experiment (xanthophores are removed from the domain at 65 dpf and their further birth is halted). Scale bar is 500 μm. This experiment influenced our rules for melanophore death; for details see Supplementary Note 1. Images in (a) are reproduced from Parichy and Turner[4] with permission of The Company of Biologists, Ltd.

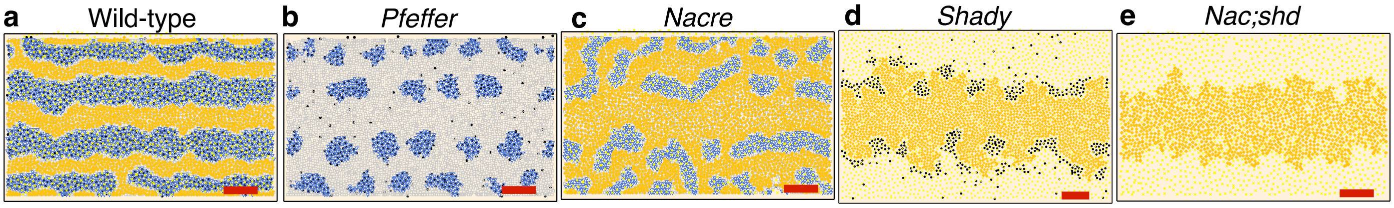

Supplementary Figure 17: Alternative xanthophore form-change mechanism. (a) Wild-type. When we remove the local signal from X^{d} to X^{l} to become dense (by setting $p = 0$ in Equation 2; also see Fig. 3e in the main text), there is necessarily no difference in (b) *pfeffer*, and (c) *nacre* and wild-type are not negatively impacted. This change is visible only in (d) *shady* and (e) *nacre*;*shady*: without a signal from dense to loose xanthophores, these mutants feature a mixture of xanthophores of both types, possibly related to the work[6], published post model development. Scale bars are 500 μm.

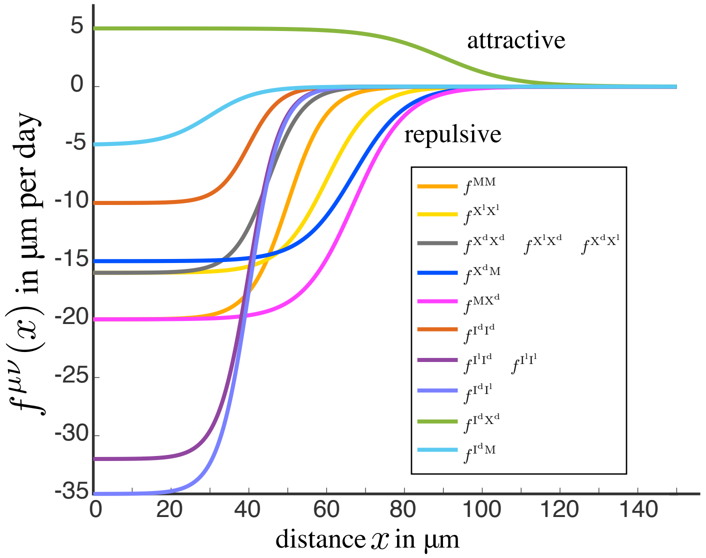

Supplementary Figure 18: Plot of pairwise migration forces. Note all forces decay to 0 quickly, modeling migration as due to cell or dendrite contact. With one exception, all cells move away from each other; we specify that I^{d} weakly attract X^{d}. Migration parameter values for all simulations: R^{MM} = 20 μm day^{-1}, $R^{\mathrm{X^lX^l}}$ = 16 μm day^{-1}, $R^{\mathrm{X^dX^d}}$ = 16 μm day^{-1}, $R^{\mathrm{X^lX^d}}$ = 16 μm day^{-1}, $R^{\mathrm{X^dX^l}}$ = 16 μm day^{-1}, $R^{\mathrm{X^dM}}$ = 15 μm day^{-1}, $R^{\mathrm{MX^d}}$ = 20 μm day^{-1}, $R^{\mathrm{I^dI^d}}$ = 10 μm day^{-1}, $R^{\mathrm{I^lI^d}}$ = 32 μm day^{-1}, $R^{\mathrm{I^lI^l}}$ = 32 μm day^{-1}, $R^{\mathrm{I^dI^l}}$ = 35 μm day^{-1}, $R^{\mathrm{I^dX^d}}$ = -5 μm day^{-1}, and $R^{\mathrm{I^dM}}$ = 5 μm day^{-1}; r_{MM} = 50 μm, $r_{\mathrm{X^lX^l}}$ = 60 μm, $r_{\mathrm{X^dX^d}}$ = 45 μm, $r_{\mathrm{X^lX^d}}$ = 45 μm, $r_{\mathrm{X^dX^l}}$ = 45 μm, $r_{\mathrm{X^dM}}$ = 67 μm, $r_{\mathrm{MX^d}}$ = 67 μm, $r_{\mathrm{I^dI^d}}$ = 40 μm, $r_{\mathrm{I^lI^d}}$ = 40 μm, $r_{\mathrm{I^lI^l}}$ = 40 μm, $r_{\mathrm{I^dI^l}}$ = 40 μm, $r_{\mathrm{I^dX^d}}$ = 90 μm, and $r_{\mathrm{I^dM}}$ = 30 μm; $\delta = 5$ μm.

Supplementary Table 1: Length scales present in our cell differentiation, death, and form change rules

**Name**	**Description**	**Source/Motivation**
$\Omega^{\mathbf{z}}_{\text{long}}$	Annulus with outer radius 250 μm and inner radius 210 μm centered at $\mathbf{z}$	Chosen on the order of half a stripe width
$B^{\mathbf{z}}_{90}$	Disk of radius 90 μm centered at $\mathbf{z}$ accounts for short-range communication at light-dark boundaries	Chosen slightly larger than $\Delta_{\text{xm}} = 82$ μm, the average distance between melanophores and xanthophores at stripe-interstripe boundaries [12]
$B^{\mathbf{z}}_{75}$	Disk of radius 75 μm centered at $\mathbf{z}$	Chosen slightly less than Δ_{xm}
$B^{\mathbf{z}}_{45}$	Disk of radius 45 μm centered at $\mathbf{z}$	Chosen to account for local communication, possibly between cells in different layers on the fish skin
$B^{\mathbf{z}}_{\Delta_{\text{xm}}}$	Disk of radius Δ_{xm} = 82 μm [12]	Appears only in cell birth overcrowding conditions

Supplementary Table 2: Cell birth parameters

Name	Value	Description	Source/Motivation
N_{diff}	350	Number of random locations selected each day for possible melanophore birth	Reproduces dynamics
$N_{\text{init}}^{\text{M}}$	40	Number of melanophores on the domain in our initial condition	Source of noise; rough approximation of larval melanophores
$N_{\text{init}}^{\text{X}}$	500	Number of loose xanthophores on the domain in our initial condition	Loose xanthophores cover the fish skin[5]
N_{rand}	10	Number of random locations selected each day for possible random birth of loose xanthophores (only operates at low densities)	Reproduces dynamics
α	3	Lower bound on $\sum_{i=1}^{N_{\text{X}}^{\text{d}}} \mathbb{1}_{\Omega^{\mathbf{z}}_{\Delta_{\text{long}}}}(\mathbf{X}_i^{\text{d}}) + \sum_{i=1}^{N_{\text{I}}^{\text{d}}} \mathbb{1}_{\Omega^{\mathbf{z}}_{\Delta_{\text{long}}}}(\mathbf{I}_i^{\text{d}}) - \beta \sum_{i=1}^{N_{\text{M}}} \mathbb{1}_{\Omega^{\mathbf{z}}_{\Delta_{\text{long}}}}(\mathbf{M}_i)$ for M differentiation at $\mathbf{z}$	Introduces a time delay between X^{d} and I^{d} arrival and M birth
β	3.5	Lower bound on $\frac{\sum_{i=1}^{N_{\text{X}}^{\text{d}}} \mathbb{1}_{\Omega^{\mathbf{z}}_{\text{long}}}(\mathbf{X}_i^{\text{d}}) + \sum_{i=1}^{N_{\text{I}}^{\text{d}}} \mathbb{1}_{\Omega^{\mathbf{z}}_{\text{long}}}(\mathbf{I}_i^{\text{d}}) - \alpha}{\sum_{i=1}^{N_{\text{M}}} \mathbb{1}_{\Omega^{\mathbf{z}}_{\text{long}}}(\mathbf{M}_i)}$ for M differentiation at $\mathbf{z}$	Same value used in[14]
η	4	Upper bound on $\sum_{i=1}^{N_{\text{X}}^{\text{d}}} \mathbb{1}_{B^{\mathbf{z}}_{\Delta_{\text{xm}}}}(\mathbf{X}_i^{\text{d}}) + \sum_{i=1}^{N_{\text{M}}} \mathbb{1}_{B^{\mathbf{z}}_{\Delta_{\text{xm}}}}(\mathbf{M}_i) + \sum_{i=1}^{N_{\text{I}}^{\text{d}}} \mathbb{1}_{B^{\mathbf{z}}_{\Delta_{\text{xm}}}}(\mathbf{I}_i^{\text{d}})$ for M differentiation at $\mathbf{z}$	Chosen based on cell-cell distance measurements[12]
ϕ	6	Upper bound on $\sum_{j=1}^{N_{\text{X}}^{\text{d}}} \mathbb{1}_{B^{\mathbf{X}_i^{\text{d}}}_{\Delta_{\text{xm}}}}(\mathbf{X}_j^{\text{d}}) + \sum_{j=1}^{N_{\text{X}}^{\text{l}}} \mathbb{1}_{B^{\mathbf{X}_i^{\text{d}}}_{\Delta_{\text{xm}}}}(\mathbf{X}_j^{\text{l}})$ for X^d differentiation from $\mathbf{X}_i^{\text{d}}$	Chosen based on cell-cell distance measurements[12]
ψ	1	Upper bound on $\sum_{j=1}^{N_{\text{X}}^{\text{d}}} \mathbb{1}_{B^{\mathbf{X}_i^{\text{l}}}_{\Delta_{\text{xm}}}}(\mathbf{X}_j^{\text{d}}) + \sum_{j=1}^{N_{\text{X}}^{\text{l}}} \mathbb{1}_{B^{\mathbf{X}_i^{\text{l}}}_{\Delta_{\text{xm}}}}(\mathbf{X}_j^{\text{l}})$ for X^{l} differentiation from $\mathbf{X}_i^{\text{l}}$	Chosen based on cell-cell distance measurements
ρ	7	Upper bound on $\sum_{j=1}^{N_{\text{I}}^{\text{d}}} \mathbb{1}_{B^{\mathbf{x}}_{\Delta_{\text{xm}}}}(\mathbf{I}_j^{\text{d}}) + \sum_{j=1}^{N_{\text{I}}^{\text{d}}} \mathbb{1}_{B^{\mathbf{x}}_{\Delta_{\text{xm}}}}(\mathbf{I}_j^{\text{l}})$ for I^{d} or I^{l} differentiation ($\mathbf{x} = \mathbf{I}_i^{\text{d}}$ or $\mathbf{I}_i^{\text{l}}$, respectively)	Chosen because iridophores proliferate more and are smaller than other cells
γ	0	Appears in conditions for random melanophore and loose xanthophore birth	Birth due to noise occurs only in empty regions

Supplementary Table 3: Melanophore death parameters

Name	Value	Description	Source/Motivation
μ	1.25	Lower bound on $\dfrac{\sum_{j=1}^{N_X^d} \mathbb{1}_{B_{90}^{\mathbf{M}_i}}(\mathbf{X}_j^d)}{\sum_{j=1}^{N_M} \mathbb{1}_{B_{90}^{\mathbf{M}_i}}(\mathbf{M}_j)}$ for cell death at $\mathbf{M}_i$	Short-range competition[3]
ξ	2	Lower bound on $\dfrac{\sum_{j=1}^{N_M} \mathbb{1}_{\Omega_{\text{long}}^{\mathbf{M}_i}}(\mathbf{M}_j)}{\sum_{j=1}^{N_X^d} \mathbb{1}_{\Omega_{\text{long}}^{\mathbf{M}_i}}(\mathbf{X}_j^d)}$ for cell death at $\mathbf{M}_i$	Long-range survival signals[3]
η	3	Upper bound on $\sum_{j=1}^{N_I^d} \mathbb{1}_{B_{45}^{\mathbf{M}_i}}(\mathbf{I}_j^l)$ for cell death at $\mathbf{M}_i$	Set to account for temperature upshift experiments[4]
p_{death}	0.0333	Probability of random M death	Based on[3]

Supplementary Table 4: Parameters in cell form rules

Name	Value*	Description**
a	2	Appears in xanthophore dense-to-loose form transition rule
b	1	Appears in xanthophore loose-to-dense form transition rule
c	3	Appears in iridophore loose-to-dense form transition rule [Ã]
d	9	Appears in iridophore loose-to-dense form transition rule [B̃]
e	3	Appears in iridophore loose-to-dense form transition rule [C̃]
f	3	Appears in iridophore dense-to-loose form transition rule [A]
g	5	Appears in iridophore dense-to-loose form transition rule [B]
h	2	Appears in iridophore dense-to-loose form transition rule [C]
p	0.5	Probability of dense xanthophores affecting xanthophore loose-to-dense transitions

*We expect these parameters could be rescaled and stress that it is the structure of our form change rules, not the parameters in them, that we suggest have biological value. **See Fig. 5 in the main text for the mechanism given by [letter] each parameter corresponds to.

Supplementary References

[1] Frohnhöfer, H. G., Krauss, J., Maischein, H. M. & Nüsslein-Volhard, C. Iridophores and their interactions with other chromatophores are required for stripe formation in zebrafish. *Development* **140**, 2997–3007 (2013).

[2] Yamaguchi, M., Yoshimoto, E. & Kondo, S. Pattern regulation in the stripe of zebrafish suggests an underlying dynamic and autonomous mechanism. *Pro. Natl. Acad. Sci. USA* **104**, 4790–4793 (2007).

[3] Nakamasu, A., Takahashi, G., Kanbe, A. & Kondo, S. Interactions between zebrafish pigment cells responsible for the generation of Turing patterns. *Pro. Natl. Acad. Sci. USA* **106**, 8429–8434 (2009).

[4] Parichy, D. M. & Turner, J. M. Temporal and cellular requirements for fms signaling during zebrafish adult pigment pattern development. *Development* **130**, 817–833 (2003).

[5] Mahalwar, P., Walderich, B., Singh, A. P. & Nüsslein-Volhard, C. Local reorganization of xanthophores fine-tunes and colors the striped pattern of zebrafish. *Science* **345**, 1362–1364 (2014).

[6] Mahalwar, P., Singh, A. P., Fadeev, A., Nüsslein-Volhard, C. & Irion, U. Heterotypic interactions regulate cell shape and density during color pattern formation in zebrafish. *Biol. Open* **5**, 1680–1690 (2016).

[7] Patterson, L. B. & Parichy, D. M. Interactions with iridophores and the tissue environment required for patterning melanophores and xanthophores during zebrafish adult pigment stripe formation. *PLoS Genet.* **9** (2013). DOI 10.1371/journal.pgen.1003561.

[8] Patterson, L. B., Bain, E. J. & Parichy, D. M. Pigment cell interactions and differential xanthophore recruitment underlying zebrafish stripe reiteration and *Danio* pattern evolution. *Nat. Commun.* **5** (2014). DOI 10.1038/ncomms6299.

[9] Singh, A. P., Schach, U. & Nüsslein-Volhard, C. Proliferation, dispersal and patterned aggregation of iridophores in the skin prefigure striped colouration of zebrafish. *Nat. Cell Biol.* **16**, 604–611 (2014).

[10] Parichy, D. M., Elizondo, M. R., Mills, M. G., Gordon, T. N. & Engeszer, R. E. Normal table of postembryonic zebrafish development: staging by externally visible anatomy of the living fish. *Dev. Dyn.* **238**, 2975–3015 (2009).

[11] Singh, A. P. & Nüsslein-Volhard, C. Zebrafish stripes as a model for vertebrate colour pattern formation. *Curr. Biol.* **25**, R81–R92 (2015).

[12] Takahashi, G. & Kondo, S. Melanophores in the stripes of adult zebrafish do not have the nature to gather, but disperse when they have the space to move. *Pigment Cell Melanoma Res.* **21**, 677–686 (2008).

[13] Parichy, D. M. & Turner, J. M. Zebrafish *puma* mutant decouples pigment pattern and somatic metamorphosis. *Dev. Biol.* **256**, 242–257 (2003).

[14] Volkening, A. & Sandstede, B. Modelling stripe formation in zebrafish: an agent-based approach. *J. R. Soc. Interface* **12** (2015). DOI 10.1098/rsif.2015.0812.

[15] Hamada, H. *et al.* Involvement of delta/notch signaling in zebrafish adult pigment stripe patterning. *Development* **141**, 318–324 (2014).

[16] Fadeev, A., Krauss, J., Singh, A. P. & Nüsslein-Volhard, C. Zebrafish leucocyte tyrosine kinase controls iridophore establishment, proliferation and survival. *Pigment Cell Melanoma Res.* **29**, 284–296 (2016).

[17] Walderich, B., Singh, A. P., Mahalwar, P. & Nüsslein-Volhard, C. Homotypic cell competition regulates proliferation and tiling of zebrafish pigment cells during colour pattern formation. *Nat. Commun.* **7** (2016). DOI 10.1038/ncomms11462.

[18] Parichy, D. M., Turner, J. M. & Parker, N. B. Essential role for *puma* in development of postembryonic neural crest-derived cell lineages in zebrafish. *Dev. Biol.* **256**, 221–241 (2003).

[19] Watanabe, M. & Kondo, S. Is pigment patterning in fish skin determined by the Turing mechanism? *Trends Genet.* **31**, 88–96 (2015).

[20] Budi, E. H., Patterson, L. B. & Parichy, D. M. Post-embryonic nerve-associated precursors to adult pigment cells: genetic requirements and dynamics of morphogenesis and differentiation. *PLoS Genet.* **7** (2011). DOI 10.1371/journal.pgen.1002044.

[21] Dooley, C. M., Mongera, A., Walderich, B. & Nüsslein-Volhard, C. On the embryonic origin of adult melanophores: the role of erbb and kit signalling in establishing melanophore stem cells in zebrafish. *Development* **140**, 1003–1013 (2013).

[22] McMenamin, S. K., Chandless, M. N. & Parichy, D. M. Working with zebrafish at postembryonic stages. *Methods Cell Biol.* **134**, 587–607 (2016).
