# Supplementary material for: Iridophores as a source of robustness in zebrafish stripes and variability in Danio patterns
Source: Nat Commun. 2018 Aug 13;9:3231. doi: 10.1038/s41467-018-05629-z (PMC6089994; doi:10.1038/s41467-018-05629-z)
Supplement: Supplementary file 3 — Description of Additional Supplementary Files [file 41467_2018_5629_MOESM3_ESM.pdf]

Description of Additional Supplementary Files

File Name: Supplementary Movie 1
Description: Wild-type development, example 1. Simulation of wild-type pattern formation by day, beginning at 21 dpf (stage PB) and ending at approximately 71 dpf (stage J+). Pattern forms autonomously due to cell interactions from the initial condition. Scale bar is 500 µm. Domain size accounts for the full width of the fish body and roughly one third of its length.

File Name: Supplementary Movie 2
Description: Wild-type development, example 2. A second example simulation of wild-type pattern formation by day, beginning at 21 dpf (stage PB) and ending at approximately 71 dpf (stage J+). Scale bar is 500 µm. Domain size accounts for the full width of the fish body and roughly one third of its length.

File Name: Supplementary Movie 3
Description: *Pfeffer* development. Example simulation of *pfeffer* pattern formation by day, beginning at 21 dpf (stage PB) and ending at approximately 71 dpf (stage J+). Scale bar is 500 µm. Domain size accounts for the full width of the fish body and roughly one third of its length.

File Name: Supplementary Movie 4
Description: *Nacre* development. Example simulation of *nacre* pattern formation by day, beginning at 21 dpf (stage PB) and ending at approximately 71 dpf (stage J+). Scale bar is 500 µm. Domain size accounts for the full width of the fish body and roughly one third of its length.

File Name: Supplementary Movie 5
Description: *Shady* development. Example simulation of *shady* pattern formation by day, beginning at 21 dpf (stage PB) and ending at approximately 80 dpf (stage J+). Note a central strip of X^d is introduced to the domain at 22 dpf. Scale bar is 500 µm. Domain size accounts for the full width of the fish body and roughly one third of its length.

File Name: Supplementary Movie 6
Description: *Choker* development. Example simulation of *choker* pattern formation by day, beginning at 21 dpf (stage PB) and ending at approximately 81 dpf (stage J+). Note 15 dense iridophores are introduced to the domain at randomly selected locations at 31 dpf. Scale bar is 500 µm. Domain size accounts for the full width of the fish body and roughly one third of its length.

File Name: Supplementary Movie 7
Description: Ablation of melanophores and xanthophores. Simulation of Experiment 1 by Yamaguchi *et al*. [4]. Wild-type pattern formation begins normally, but at 40 dpf we remove all melanophores, all dense and loose xanthophores, and 20% (randomly selected) of dense and loose iridophores in a central 1000 µm-long region across the full height of the domain. After this perturbation, the simulation continues without further disruption; by 80 dpf (stage J+), the stripe pattern has regenerated with lost directionality but maintained width.

Scale bar is 500 µm. Domain size accounts for the full width of the fish body and roughly one third of its length.
